# Profiling expression changes caused by a segmental aneuploid in maize

**DOI:** 10.1186/1471-2164-9-7

**Published:** 2008-01-10

**Authors:** Irina Makarevitch, Ronald L Phillips, Nathan M Springer

**Affiliations:** 1 Microbial and Plant Genomics Institute, Department of Plant Biology, University of Minnesota, St. Paul MN 55108, USA; 2 Microbial and Plant Genomics Institute, Department of Agronomy and Plant Genetics, University of Minnesota, St. Paul MN 55108, USA; 3 Biology Department, Hamline University, St. Paul MN, USA

## Abstract

**Background:**

While changes in chromosome number that result in aneuploidy are associated with phenotypic consequences such as Down syndrome and cancer, the molecular causes of specific phenotypes and genome-wide expression changes that occur in aneuploids are still being elucidated.

**Results:**

We employed a segmental aneuploid condition in maize to study phenotypic and gene expression changes associated with aneuploidy. Maize plants that are trisomic for 90% of the short arm of chromosome 5 and monosomic for a small distal portion of the short arm of chromosome 6 exhibited a phenotypic syndrome that includes reduced stature, tassel morphology changes and the presence of knots on the leaves. The knotted-like homeobox gene *knox10*, which is located on the short arm of chromosome 5, was shown to be ectopically expressed in developing leaves of the aneuploid plants. Expression profiling revealed that ~40% of the expressed genes in the trisomic region exhibited the expected 1.5 fold increased transcript levels while the remaining 60% of genes did not show altered expression even with increased gene dosage.

**Conclusion:**

We found that the majority of genes with altered expression levels were located within the chromosomal regions affected by the segmental aneuploidy and exhibits dosage-dependent expression changes. A small number of genes exhibit higher levels of expression change not predicted by the dosage, or display altered expression even though they are not located in the aneuploid regions.

## Background

Organisms with a chromosome number that is not an even multiple of the haploid chromosome number are termed aneuploids. Alterations in the dosage of portions of a chromosome, such as the presence of an extra arm of a chromosome, are referred to as segmental aneuploidy. Changes in chromosome number frequently lead to phenotypic consequences. Some of the earliest studies of genetic mutants actually involved chromosomal alterations instead of point mutations (reviewed by [1]). Analysis of a complete set of maize monosomic lines reveals that most monosomic lines display a reduction in stature; additionally, specific phenotypes are associated with each monosomic condition [2]. Trisomic lines have been widely used for mapping in many plant species and numerous phenotypes have been associated with certain chromosomes [3-5]. In humans, aneuploidy for most chromosomes results in lethality. Aneuploidy is only tolerated for chromosome 21 (Down syndrome) and the sex chromosomes (Turner or Klinefelter syndromes). Most of the cancerous somatic human cells have been shown to carry different aneuploid chromosome sets (reviewed by [6,7]). Despite the widespread interest in aneuploidy we still have a limited understanding of the molecular mechanisms that lead to phenotypic alterations in aneuploid organisms.

Various models have been proposed to explain the phenotypic consequences caused by aneuploidy. The simplest model suggests that all genes in the aneuploid region will display increased or decreased transcript levels in direct proportion with their structural dosage and that the dosage imbalance of genes on the altered chromosome relative to genes located on other chromosomes leads to phenotypic consequences. A second, non-mutually exclusive, model posits that slight alterations in the expression level of transcription factors, or other regulatory proteins, might result in perturbations of regulatory networks such that genes located throughout the genome may be up- or down-regulated. Other models have suggested complex interactions that might result in drastic alterations of expression levels. Some of the major questions regarding gene expression and aneuploidy are: (1) what proportion of genes within the affected regions exhibit dosage dependent alterations in expression levels, (2) how common is altered expression for genes located on chromosomes not involved in the aneuploidy and (3) how common are expression changes that are more severe than the dosage alteration would predict.

Several studies have examined gene expression patterns in maize aneuploids. Alterations in the genomic dosage of the *Adh1 *gene from 1 to 4 copies do not result in concordant changes to the expression level [8]. This is likely due to the presence of a negative regulator on the same chromosome such that the balance of the regulatory and *Adh1 *genes is constant as chromosome dosage changes [9]. Guo and Birchler [10] used fourteen B-A translocation stocks to study the effects of aneuploidy on the expression of six maize genes. Three of the genes, *Adh1, Adh2 *and *Glb1*, did not show any significant change in gene expression in either a monosomic or trisomic state, suggesting the occurrence of dosage compensation. Interestingly, the expression level of all six of these genes was often sensitive to dosage of other regions and showed tissue-specific differences. For example, when the dosage of the long arm of chromosome 7 was increased, the expression of *Adh1 *and *Sus1 *expression in the embryo decreased while endosperm expression of *Glb1 *and *Zein *increased even though none of these genes are located on chromosome 7. Gene expression levels are much more sensitive to aneuploid changes than they are to changes in ploidy level [11]. Similarly, a study of trisomic tomato plants found evidence for dosage dependent expression changes for 3 of 4 genes tested [3].

The effects of aneuploidy on gene expression have also been examined in humans and mice. There have been conflicting reports on the frequency of genes that exhibit expression changes in individuals trisomic for chromosome 21 [12-14]). In some studies of multiple tissues there was evidence for global up-regulation of genes on chromosome 21 in patients with trisomic 21 [12,13]. However, another study found that only ~30% of the expressed genes on chromosome 21 exhibit altered expression level [14]. These differences might be due to difficulties in accurately monitoring small changes (~1.5-fold) in gene expression. Studies of mouse models for Down syndrome have found that many of the genes that are located in the trisomic regions display a 50% increase in expression level [15,16]. The general consensus from studies of mammalian trisomics has been that many of the genes with altered expression map to the trisomic chromosome but that there are also examples of genes in other genomic locations with altered expression levels.

In this study we have monitored the phenotypic alterations and gene expression changes that occur in a maize segmental aneuploid. These lines were initially developed in an attempt to create a genetic male sterility system for maize [17]. This segmental aneuploid carries three copies for most of the short arm of chromosome 5 and displays a phenotypic syndrome that includes leaf development alteration, delayed flowering and floral morphology changes.

## Results

### Segmental aneuploid plants derived from T5-6b display phenotypic abnormalities

**Figure 1:**
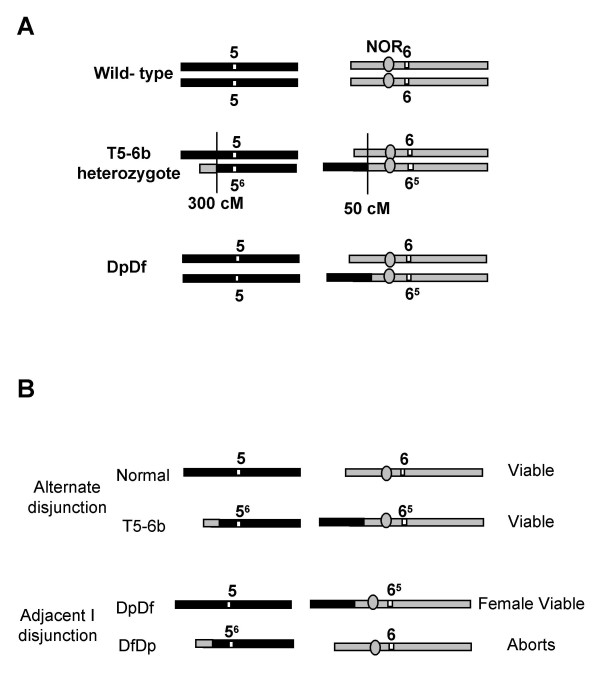
**Schematic presentation of the chromosomes 5 and 6 in DpDf plants**. (A) The chromosome 5 and 6 constitution for wild-type, T5-6b heterozygous, and DpDf plants is indicated. The gray shading is used to indicate chromosome 6 while the black shading indicates chromosome 5. The white box indicates the approximate position of the centromere while the gray oval indicates the position of the NOR. The lines indicate the position of the chromosomal breaks involved in the translocation and the cM indicates the approximate genetic position of the normal chromosome that is involved in the translocation. (B) Chromosome 5 and 6 constitution in gametes produced by alternate or adjacent I disjunction in a T5-6b heterozygote. The viability of gametes containing these chromosomal constitutions is indicated to the right of the chromosomes.

**Figure 2:**
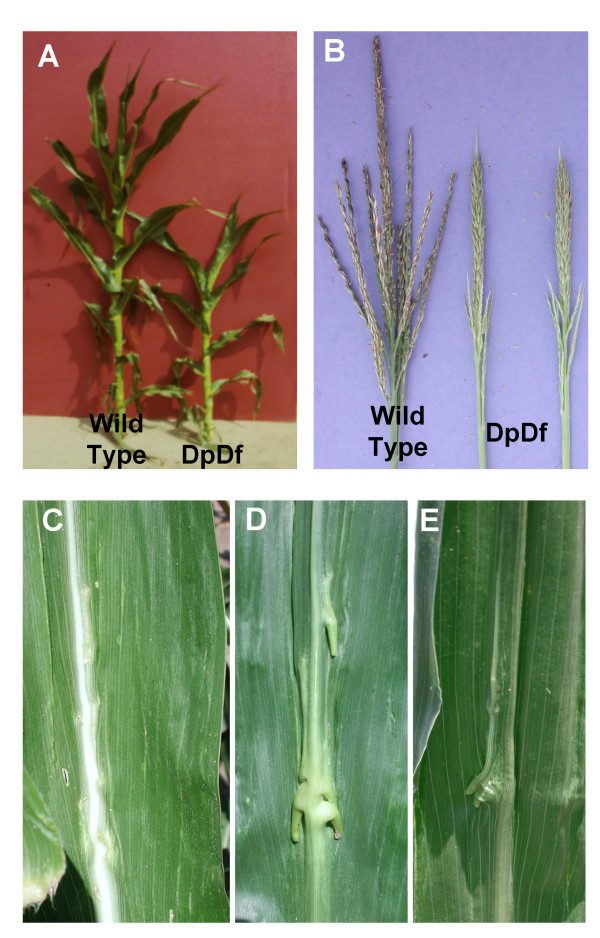
**Phenotypic effects of DpDf segmental aneuploidy in maize**. (A) DpDf plants are smaller and are ca. 2 weeks behind in development. (B) DpDf plants display partially necrotic tassels that are shorter and have fewer branches. The main rachis of the tassel tends to be thicker than in wild-type siblings. (C-E) show views of the knots that form on the leaves of DpDf plants. The adaxial surface of the leaf blade (C) shows ectopic ligule formation near the midrib while the abaxial surface (D and E) display knot-like protrusions.

Adjacent I disjunction of a plant that is heterozygous for T5-6b (Figure 1) can result in the production of a viable duplicate-deficient (DpDf) gamete. This gamete, which has a duplication for the majority of the short arm of chromosome 5 and is deficient for a small region of chromosome 6, is female transmissible but can not be transmitted through the male parent [17]. The fertilization of this female DpDf gamete by a normal male gamete results in the production of a plant with segmental aneuploidy. These plants contain three copies of the majority of the short arm of chromosome 5 and only one copy of a small portion of the short arm of chromosome 6 (Figure 1) and are referred herein as DpDf. The DpDf plants display a phenotypic syndrome that includes leaf knotting, partial tassel sterility, late flowering time and overall changes in the plant architecture (Figure 2, Table 1). DpDf plants are shorter than their wild-type siblings and have smaller stalk diameter (Table 1). The flowering time of DpDf plants is delayed 8–13 days relative to their wild-type siblings. The tassels of DpDf plants are shorter and thicker compared to wild-type siblings and have a smaller number of branches. Notably, the tassels of DpDf plants are partially necrotic and produce many branches with few to no developed florets (Figure 2, Table 1). The phenotypic syndrome of DpDf plants is more severe in DpDf plants produced from self-pollinated DpDfs (Table 1) than in the first generation DpDf plants, suggesting the accumulation of phenotypic effects when the segmental aneuploid condition is maintained for multiple generations. To understand the molecular basis of the other phenotypic alterations in the DpDf plants, we used a more comprehensive approach and looked at changes in gene transcription levels caused by segmental aneuploidy.

**Table 1:** Phenotypic syndrome present in DpDf plants.

Trait	DpDf -2nd generation (100 plants)	DpDf -1st generation (100 plants)	Wild-type (100 plants)
Height (cm)	192 +/- 13^a^	198 +/- 14^a^	212 +/- 9^b^
Stalk diameter (cm)	18.9 +/- 2.4^a^	21.2 +/- 2.5^b^	23.6 +/- 2.1^c^
Knot severity (scale 0 to 5)	4.5 +/- 1.1^a^	2.4 +/- 0.9^b^	0.0 +/- 0.0^c^
Tassel diameter (mm)	15 +/- 2^a^	15 +/- 3^a^	9 +/- 1^b^
Number of tassel branches	8.7 +/- 2.0^ab^	7.7 +/- 2.1^b^	9.1 +/- 1.6^a^
Tassel height (mm)	316 +/- 46^a^	354 +/- 48^b^	428 +/- 25^c^
Tassel viability (scale 0 to 5)	3.5 +/- 1.4^a^	4.4 +/- 0.7^b^	4.9 +/- 0.3^c^

Values denoted by different subscript letters are statistically significant at p < 0.001.

### Aneuploidy causes limited changes at the transcript level

**Figure 3:**
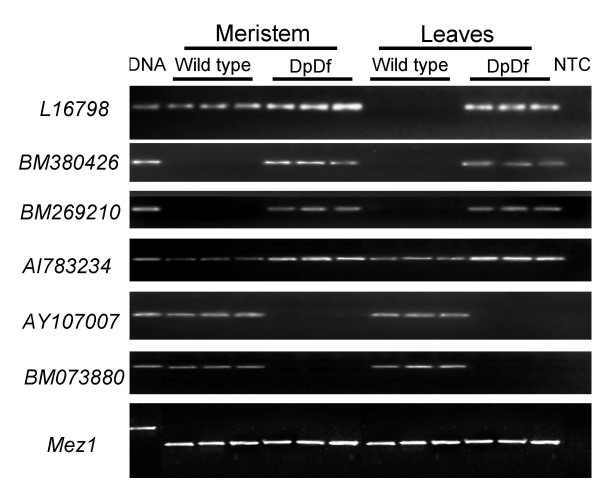
**Validation of severe expression changes detected by expression profiling**. For six of the genes with the greatest alteration in gene expression changes, the expression levels were validated in two tissue types from three different wild type and three different DpDf sibling plants by semi-quantitative RT-PCR. L16798, BM380426, BM269210 and AI783234 are examples of increased expression in DpDf plants while AY107007 and BM073880 are examples of decreased expression in DpDf plants. *Mez1 *is used as a loading control. The first lane is a genomic DNA control while the last lane is a no template control (NTC).

The altered gene dosage in a segmental aneuploid is expected to result in a dosage-dependent change in gene expression for all genes within the affected regions. Therefore, it is expected that many of the expressed genes on the duplicated region of 5S will show 1.5 fold increase in transcript level relative to wild-type siblings while genes on the deficient portion of chromosome 6 will mostly show 0.5 fold decrease in transcript levels relative to wild-type siblings. Due to gene interactions and regulation networks, it is also possible that alteration in the transcription levels of certain transcription factors might affect transcriptional networks and result in altered expression of genes located throughout the genome. We interrogated maize Affymetrix microarrays containing probes for ca. 14,000 genes with RNA samples from DpDf and wild-type plants to study the effects of segmental aneuploidy on the maize transcriptome. In this study we investigated gene transcript levels and use the word "expression" to refer to the abundance of RNA transcripts, not proteins. Although the most severe phenotypic abnormalities were observed in floral and mature leaf tissues, the developmental delay in DpDf plants relative to wild-type siblings complicated our ability to perform controlled sampling of these tissues for microarray studies. Therefore, we chose to sample 11-day old seedlings as these tissues display very little phenotypic difference between wild-type and DpDf siblings and there are few developmental transitions occurring at this phase of vegetative growth. At the 11-day seedling stage it is not possible to distinguish between DpDf plants and wild-type siblings without molecular marker analyses. Pooled RNA samples were prepared from four biological replicates from DpDf plants and their wild-type siblings and used for microarray hybridization. A series of statistical tests, ratio cut-offs and expression level criteria were applied to identify a set of 596 differentially expressed genes ([see Additional file 1]; see Materials and Methods for detailed explanation of the statistical analysis and filtering criteria). We found that most (83%) of the differentially expressed genes exhibit increased transcript levels in DpDf plants relative to wild-type siblings (Table 2). In conformity with an expected dosage-dependent change in expression, the vast majority (579/596) of the differentially expressed genes exhibited less than a two-fold change in transcript levels in wild-type relative to DpDf plants. Analysis of the GO annotations for the differentially expressed genes did not reveal evidence for over-representation of any functional categories (Figure 3).

**Table 2:** Analysis of genes differentially expressed in DpDf plants compared to wild-type

	Total number of genes	Genes with predicted or determined map positions	Genes mapped to trisomic portion of 5S	Genes mapped to deleted portion of 6
Increased transcript in DpDf	496	390	304	0
10–20 fold change	3	3	2	0
2–2.6 fold change	8	7	6	0
1.2–2 fold change	485	380	296	0
				
Decreased transcript in DpDf	100	68	4	8
2–2.2 fold change	6	6	1	5
1.2–2 fold change	94	62	3	3
				
Total number of genes	596	458	308	8

**Figure 4:**
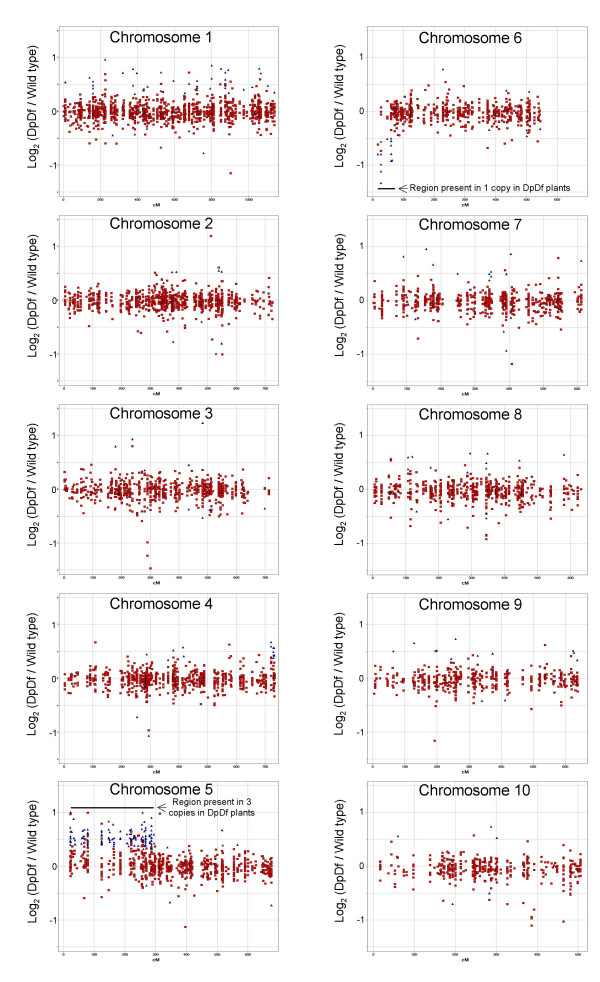
**Genome distribution of the genes with altered expression levels in DpDf plants**. Using a bioinformatic approach, we were able to infer the map positions for ~7,000 microarray probe sets. The probe sets were divided into the 10 separate plots (one for each maize chromosome). The log_2 _of the average DpDf microarray signal divided by the average wild-type microarray signal is plotted on the y-axis and the cM (based on IBM 2004 coordinates) is plotted along the x-axis. A log_2 _value of 0 corresponds to equal expression levels in DpDf and wild type. A log_2 _value of 1.0 indicates two-fold higher expression in DpDf than in wild-type while a log_2 _value of -1.0 indicates two-fold lower expression in DpDf than in wild-type. The blue triangles indicate genes that were found to have statistically significant differential expression in DpDf and wild-type plants (and passed the minimum signal and fold-change filters). The red squares indicate genes that were not deemed to have statistically significant differential expression. The regions of chromosome 5 and 6 that are present in altered copy number are indicated by black bars.

Seventeen of the differentially expressed genes are more than 2-fold changed in comparisons of DpDf and wild-type plants (Table 3). These included 11 examples of up-regulation in DpDf plants and six examples of down-regulation in DpDf plants. We confirmed expression change by RT-PCR and determined the chromosomal location for fifteen of these seventeen genes using identity with mapped maize BAC contigs and experimentally by mapping with the oat × maize chromosome addition lines [18]. Eight of the ten genes that displayed greater than 2-fold increased transcript levels in DpDf plants mapped to chromosome 5 (Table 3). The other two genes mapped to chromosomes 1 and 8. Each of the five down-regulated genes that were tested mapped to the distal portion of chromosome 6S, probably in the monosomic region. RT-PCR was used to confirm the expression differences observed using microarrays. Interestingly, for 6 of 10 up-regulated genes and for all 5 down-regulated genes that were tested, we observed transcripts in only wild-type or only aneuploid plants (Table 3). In each of these cases we were only able to detect transcripts in either wild-type or DpDf plants following 35 cycles of PCR. The other four up-regulated genes showed evidence of expression in both DpDf and wild-type with quantitative variation. We proceeded to assess the expression differences in two different tissues from wild-type or DpDf individual plants (Figure 4). One of these genes, L16798, was ectopically expressed in mature leaf tissue in DpDf plants similar to the expression observed for *knox10 *(Figure 4). Another gene, AI783234, exhibited more severe up-regulation in the meristem derived tissue (Figure 4). For the majority of genes, including BM380426, BM269210, AY107007 and BM073880, both meristem and leaf tissue exhibited the altered expression patterns (Figure 4).

**Table 3:** Analysis of genes with greater than 2-fold change between DpDf and wild type siblings

Accession	Wild type (signal)	DpDf (signal)	Fold change (DpDf/Wild type)	Expression validation^a^	Chromosome^b^	Annotation
Increased transcript levels in DpDf relative to wild type						
AI666121	18	414	23.09	Present/absent	5	peptidyl-prolyl cis-trans isomerase
L16798	12	140	11.44	Present/absent	5	class I acidic chitinase
BM380426	31	339	11.10	Present/absent	1	unknown
AI783234	169	429	2.53	Semi-quantitative	5	unknown
BM269210	81	198	2.46	Present/absent	5	splicing factor Prp18
AY639019	49	119	2.41	Present/absent	8	phosphate transporter
AY105653	358	842	2.35	Semi-quantitative	5	malate oxidoreductase
CK369759	63	145	2.32	Present/absent	5	unknown
CF629635	187	375	2.01	Semi-quantitative	5	SEC14 cytosolic factor
AY107589	317	633	2.00	Semi-quantitative	5	unknown
U17897	200	415	2.07	NT^c^	NT^c^	starch branching enzyme I (sbe1)
						
Decreased transcript levels in DpDf relative to wild type						
AY107007	78	37	0.47	Present/absent	6	unknown
AI622092	200	92	0.46	Present/absent	6	unknown
BG873775	135	62	0.46	Present/absent	6	lipid transfer protein
BM073880	109	48	0.44	Present/absent	6	similar to GCN5-like protein 1
AI737202	192	76	0.40	Present/absent	6	unknown
AI677337	874	427	0.49	NT^c^	NT^c^	unknown

^a^Expression validation was assessed as "Present/absent" when amplicons were only detected in either DpDf or wild-type. Semi-quanitative indicates that expression was detected in both DpDf and wildtype with semi-quantitative variation in agreement with the microarray results.

^b^Chromosome positions determined from oat × maize chromosome addition lines (Kynast et al., 2001).

^c^Not tested due to failed amplification

### Genomic distribution of differentially expressed genes

We were interested in determining the genomic distribution of the 596 differentially expressed genes to determine whether they occurred primarily within the segmental aneuploid regions. Although the maize genome sequencing effort was still in progress, over one third of the BACs were sequenced and mapped at the time of analysis (January 2007; [19]); therefore, it was possible to determine map positions for a large subset of array features. A computational effort was implemented to map Affymetrix consensus probe sequences to maize BAC or overgo [20] sequences. In many cases the maize BACs have been characterized by HICF (High Information Content Fingerprint) and placed in contigs. Many of these contigs have been tied to the maize genetic map through the hybridization of genetically mapped sequences to the BAC clones. The implementation of this computational approach resulted in putative map positions for ~7,000 of the 13,495 genes present on the Affymetrix array [see Additional file 2]. This approach may result in obtaining incorrect map positions due to improper alignments to the wrong BAC end sequences (often complicated by presence of paralogous sequences), incorrect BAC contigs or incorrect placement of a BAC contig onto the physical map of maize. However, we validated map positions for 25 mapped features chosen at random using the oat × maize chromosome addition lines [18] and radiation hybrid oat × maize lines [21] and found that 24 of the 25 features were correctly mapped to the proper chromosome region using our computational approach. Applying these rates, we would predict that the computationally inferred map locations are likely to be accurate for ~95% of the ~7,000 features.

**Figure 5:**
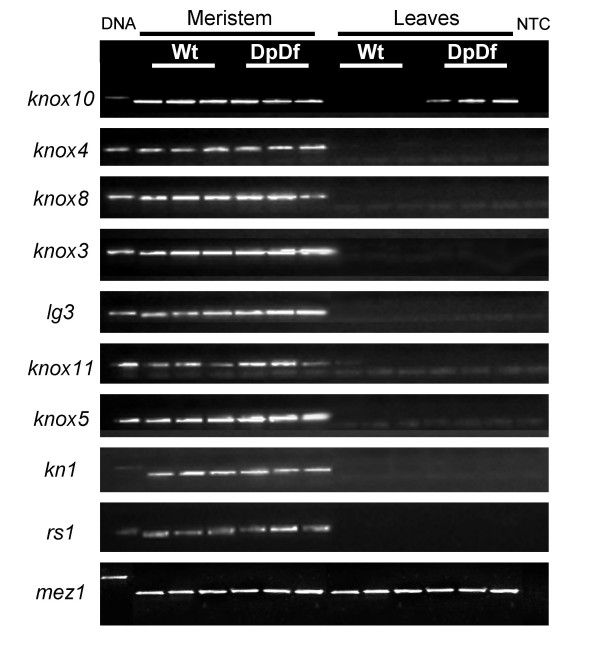
***Knox10 *gene is the only knotted-like gene ectopically expressed in leaves of DpDf plants**. Expression of nine knotted-like maize genes was tested in meristematic and leaf tissue in wild type and DpDf plants using RT-PCR. Leaf and meristematic tissue was isolated from three seedlings for each of the genotypes. *Mez1 *gene was used as a control to equalize the cDNA concentrations for all the samples. The tissue type and genotype are indicated above the gel images. The first lane is a genomic DNA control while the last lane is a no template control (NTC). Control reactions were also performed on a no reverse-transcriptase control (data not shown).

We proceeded to analyze the genome-wide distribution of the differentially expressed genes (Figure 5). Map positions were inferred for 458 of the 596 of the differentially expressed genes, including 390 genes that were up-regulated in DpDf plants and 68 genes that were down-regulated in DpDf plants. Three-fourths of the up-regulated genes (304/390) are located within the region of chromosome 5S that is present in three copies (Figure 5). The remaining 86 genes map to genomic locations that are not involved in the segmental aneuploidy, suggesting the existence of gene regulation and interaction networks. The 68 genes that are down-regulated in DpDf plants include 13 genes on chromosome 6 and most of these are likely within the region that is present in only one copy. The remaining 55 down-regulated genes are distributed throughout the genome (Figure 5).

We were interested in assessing the proportion of genes on chromosome 5S that display dosage-dependent alterations in gene expression. A set of 334 genes for which we had good positional information to suggest they were located within the trisomic region were analyzed in more detail ([see Additional file 3]; Figure 5). This list of genes was filtered to identify the 280 genes that were expressed in wild-type seedlings (over 50 units of signal for the normalized GC-RMA signals. One hundred twenty-one (43%) of these genes were identified as up-regulated in DpDf plants, 1 gene was down-regulated and 158 genes were not affected in DpDf plants. Using the oat × maize radiation hybrids we were able to validate the map position for 19/20 of these genes to chromosome 5S [see Additional file 3]. The one gene that was not validated to map to chromosome 5 appears to be a member of a multi-gene family with copies present on 5 and other maize chromosomes. Analysis of the distribution of these 280 genes on the chromosome indicated that there was no positional bias for the responsiveness to dosage (Figure 5, [see Additional file 3]). We compared the GO annotation for the genes on the region of 5S chromosome duplicated in DpDf plants that were dosage-sensitive to those that were dosage-insensitive. We did not find any evidence for statistical over-representation of any functional categories (Figure 3). We also did not notice a correlation between the expression level of a gene and its likelihood to be sensitive to dosage changes.

A second approach was used to assess the proportion of genes within the aneuploid region of chromosome 5 that exhibit dosage-dependent changes in expression level. We made the assumption that genes with no alteration in expression level in DpDf plants relative to normal siblings will exhibit a normalized distribution of ratios centered at the value of 1. If this assumption is correct then the number of genes with a DpDf:wild-type ratio less than 1 will be one half of the genes that do not exhibit dosage-dependent alterations in expression level. We found that 64 of the 280 genes exhibit a ratio less than 1. This suggests that 128 of the 280 genes (46%) do not exhibit any evidence for altered expression in the aneuploid state while the remaining 54% of genes exhibit some level of increased expression as a result of increase dosage. These frequencies are quite similar to those obtained using per-gene statistical tests for differential expression as described above. In contrast, when we assessed the distribution of all values not located on chromosome 5S or 6S we found a normalized distribution centered near 1. This suggests that aneuploidy does not have genome-wide directional affects on gene expression for regions without altered dosage.

### Alteration in expression pattern of the Knox10 gene causes leaf knotting in DpDf plants

A notable difference between DpDf plants and wild-type siblings in the B73 background is the presence of knots on the leaves (Figure 2, Table 1) that tend to cluster near the midrib, but in severely affected plants they can be found throughout the leaf blade. These knots are visible on both adaxial and abaxial surfaces of the leaf. The leaf knotting phenotype was only noticed in the B73 genetic background. When the translocation was present in other genetic backgrounds (A619, A632 or Mo17) we did not notice the knotting phenotype. The presence of knots on the leaf blade is a unique phenotypic condition widely characterized at the molecular and genetic level; thus providing us with the opportunity to study the possible causes of leaf knot formation in DpDf plants in more detail [22-24].

**Figure 6:**
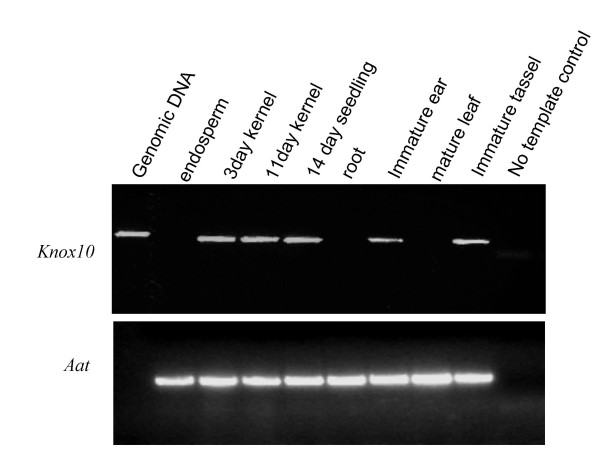
**Developmental expression pattern of the *knox10 *gene in wild-type B73 tissues**. RT-PCR was performed to detect expression of the knox10 gene in cDNA derived from 11-day post pollination endosperm, 3-day post pollination whole kernels, 11-day post pollination whole kernels, 14 day seedlings, roots, immature ear, mature leaf and immature tassel. Expression is detection in every plant tissue containing apical meristems except for roots. The lower image is a loading control showing similar levels of transcript detected in these samples using primers for the aat gene.

Knotting can be caused by ectopic expression of class 1 *knotted-like *(*knox*) genes that act as meristem-identity genes in developing leaf tissue [25-27]. A subset of the knox genes, the class I knox genes, show expression specifically in the meristem tissue [22]. However, prolonged ectopic expression of knox genes in differentiating leaf primordia is thought to lead to aberrant differentiation of some cells in the leaf blade resulting in the acquisition of sheath or ligule identity [27]. The maize genome contains at least 13 knox genes (class 1: *kn1*, *rs1*, *lg3*, *knox3*, *knox4*, *knox5*, *knox8*, *knox10*, and *knox11*; and class 2: *knox1*, *knox2*, *knox6*, and *knox7*) [22], with one gene, *knox10*, located in the trisomic region of DpDf plants (180 cM – IBM2 2004 neighbors genetic map). The *knox6 *gene is also located on chromosome 5 but proximal to the translocation breakpoint (IBM2 2004 neighbors genetic map). We investigated the expression of all nine class 1 knotted-like maize genes in the meristematic and leaf tissue of the normal and DpDf 11-day old maize seedlings by RT-PCR (Figure 6). All nine of the investigated knox genes exhibited detectable expression in meristematic tissue of both normal and DpDf plants. We were not able to detect expression for any of these nine genes in developing and mature leaf tissue of wild-type plants. In DpDf plants, ectopic expression of *knox10 *was detected in developing and mature leaf tissues while expression of the other eight genes was not detected in these tissues (Figure 6). The microarray profiling of the expression levels (see above) for the four class 2 knox genes, *knox1, knox2, knox6*, and *knox7 *showed no changes in DpDf plants compared to their normal siblings in whole seedling tissues. Therefore, only the *knox10 *gene was expressed in leaves of DpDf plants suggesting that the change in expression pattern of *knox10 *is responsible for the leaf knotting phenotype.

The *knox10 *gene is normally expressed in multiple meristematic tissues including kernels, seedlings, immature ears and tassels, but not in endosperm, roots or mature leaves (Figure 6). An analysis of the expression level of *knox10 *in meristematic tissue suggests that the expression level for this gene is slightly higher in DpDf plants than in wild-type siblings (data not shown). However, in leaf tissue, the difference between wild-type and DpDf is not a fold change but presence versus absence of the *knox10 *transcript. It is interesting that this is a potential case in which the specific level of expression may not be as critical as the altered tissue-specific pattern of expression caused by segmental aneuploidy.

## Discussion

While there is a significant amount of evidence to link aneuploidy to phenotypic syndromes or diseases, relatively little is known about the specific molecular mechanisms. In this study we documented the phenotypic alterations in a maize segmental aneuploid and studied gene expression changes. These studies allowed us to speculate on the inheritance of aneuploid syndromes, the causes of specific phenotypes in aneuploid organisms and the prevalence of expression changes that are conditioned by aneuploidy.

### Inheritance of aneuploid syndromes

One particularly interesting finding was that the severity of the aneuploid syndrome became more pronounced following multiple generations in the aneuploid state. For many of the phenotypes that were scored, a first generation DpDf plant (generated by self pollination of a heterozygous translocation individual) exhibited less severe phenotypes than a DpDf plant that was produced by an aneuploid parent. Maintaining plants in an aneuploid state for more than two generations did not result in noticeably more severe phenotypes. We are not aware of other studies in plants or animals that have assessed the severity of aneuploid syndromes in the first generation compared to later generations.

### Expression changes in a maize segmental aneuploid

The expression profiling of DpDf plants relative to euploid siblings allows us to address three important questions about aneuploidy and gene expression. First, what proportion of genes within the affected region of the genome will exhibit altered expression? Second, how common are expression changes for genes that map to other genomic locations? Third, how common are alterations of gene expression that are of greater magnitude than would be predicted by the altered dosage?

There have been reports of genes that exhibit expression changes when the dosage of the gene is altered and genes with no expression changes despite altered copy number [3,10]. Analysis of 280 expressed genes on the short arm of chromosome 5 indicates that approximately half of these genes (43% or 54% depending upon the method used) display increased expression levels in plants that are trisomic for this region. We could not detect enrichment for specific functional classifications of genes in the dosage dependent or independent gene lists. In addition, there is no evidence for chromosomal domains within the aneuploid segments that are dosage dependent or independent. It is somewhat surprising that such a large proportion of genes do not exhibit altered expression levels when the dosage of the gene is altered. This may reflect the presence of an autosomal dosage compensation system as suggested by [28].

Our data can be used to assess the relative frequency of trans-acting changes conditioned by segmental aneuploidy. Studies of both plant [10] and animal [13,29,30] aneuploids have noted expression changes for some genes that are located in regions of the genome that are not affected by the aneuploidy. This is likely attributable to perturbation of regulatory networks or altering the dosage of trans-acting factors that affect transcription. This phenomenon was also noted in our expression profiling experiments. We found that 84 of the 390 (22%) of the up-regulated genes with map positions were located in chromosomal regions not affected by the aneuploidy. The similar number of genes with increased (84) or decreased (60) transcript levels that map to positions in the genome not affected by aneuploidy suggests that the perturbations of regulatory networks by increasing the dosage of 5S is equally likely to result in up- or down-regulation of the targets. While we did certainly find evidence for regulatory networks and trans-acting effects of altered dosage it should be noted that these were relatively rare. Of the ~6,500 genes that are present on the array and map to genomic regions that are not part of the affected regions, only 2.2% (144 genes) exhibited altered expression. This may reflect a limited role for the perturbation of regulatory networks by aneuploidy or it may reflect the fact that the short arm of chromosome 5 contains relatively few factors that are involved in dosage-sensitive regulatory networks.

The majority of genes with differential expression exhibit the expected 1.5 fold change within the trisomic region. There were only a limited number of genes with expression changes in excess of the predicted 50% increase. Interestingly, many of these genes (87%) mapped to the regions involved in the segmental aneuploidy. We might have predicted that these expression changes would be the result of the perturbation of a regulatory network that might cause the down-stream targets to exhibit more severe variation. Overall, this suggests that the majority of expression changes within aneuploids are of relatively small magnitude and that perturbation of trans-acting regulatory networks rarely results in major expression level changes.

### Altered expression patterns in a maize segmental aneuploid

A particularly interesting finding was the developmental variation for altered expression patterns in the DpDf plants. For some of the genes with severe expression variation (*knox10 *in Figure 6; L16798 and AI783234 in Figure 4) the expression changes were more pronounced in specific tissues. Therefore, the presence of developmental phenotypes may be linked to differences in expression pattern, not the absolute level of gene expression. The causes of the knotted phenotypes in maize have been well characterized. The class I *knox *genes are meristem identity genes that must be repressed to allow for proper leaf initiation and development. Prolonged expression in developing leaves often results in knotting of the leaves. We found that the class I knox gene *knox10 *is not properly regulated in DpDf plants. Instead, this gene is ectopically expressed in developing leaf tissue and is the likely cause of this specific aspect of the phenotype. It is possible that many of the developmental phenotypes associated with aneuploid syndromes are the result of altered expression patterns and not altered expression levels. Further studies are necessary to characterize the exact molecular mechanisms that lead to altered expression patterns in aneuploidy.

## Conclusion

In this study we have examined the gene expression and phenotypic differences between segmental aneuploid maize plants and wild-type siblings. We found that some of the genes within the aneuploid regions exhibit dosage-dependant expression changes while other expressed genes within the aneuploid regions do not exhibit any expression differences. The finding that many expressed genes with altered genomic dosage do not exhibit expression differences suggests that dosage-compensation is occurring. Relatively few genes located in genomic regions not affected by the segmental aneuploidy exhibit altered expression levels. We found that these DpDf plants exhibit a leaf knotting phenotype that is likely caused by ectopic expression of the homeobox gene *knox10*. It is possible that many of the phenotypic abnormalities exhibited by aneuploid individuals may be the result of altered expression patterns, not altered expression levels.

## Methods

### Plant materials and tissue collection

The T5-6b translocation, backcrossed into B73 maize genetic background for over 10 generations, was available from the University of Minnesota collection. The interchange T5-6b possesses a break at 5S.1 (ca. 300 cM on IBM2 2004 Neighbors genetic map) and between the middle and distal chromomere of the satellite of 6S (break occurs prior to 50 cM on IBM2 2004 Neighbors genetic map) [18,19]. Duplicate-Deficient (DpDf) heterozygous plants were identified among progeny derived from crossing a female B73/T5-6b translocation heterozygote by a male B73 plant. The chromosome constitution of DpDf heterozygous plants is normal for all chromosomes except 5 and 6. The DpDf plants contain one normal chromosome 6 and one 6^5 ^chromosome that is lacking the terminal chromomere of the chromosome 6 satellite and contains ~90% of the short arm of chromosome 5 (Figure 1). Four biological replicates were grown using standard greenhouse conditions (1:1 mix of autoclaved field soil and MetroMix; 16 hours light and 8 hours dark; daytime temperature of 30°C and night temperature of 22°C) and sampled for gene expression on the 11th day after planting between 8:00 and 9:00 am. The plants were cut immediately above the highest brace root, thus all above-ground tissues and meristems were collected. For each biological replicate, sibling seeds produced by self pollination of a DpDf plant that segregate for wild-type and DpDf plants were planted individually and thirty plants were collected and genotyped using an SSR marker (bnlg161) that is tightly linked to the translocation breakpoint on chromosome 5. A pool of twelve wild-type plants and a separate pool of twelve DpDf plants were generated from each of the biological replicates. The sampled tissues were flash frozen in liquid nitrogen and stored at -80°C prior to RNA isolation. For *knox10 *expression studies, the plants were grown as described above and two tissue types were collected from individual plants. One sample included the shoot apical meristems and leaf primordium (approximately 1 cm of tissue from the shoot apical meristem) and the second tissue type contained developing and mature leaf tissue.

### RNA isolation and microarray hybridization

RNA isolation and Affymetrix (Santa Clara, CA, USA) microarray hybridizations were performed as described in [20] with some modifications for four biological replicates of wild-type B73 and DpDf plants. Briefly, tissues from 12 seedlings per genotype per biological replicate were pooled and ground in liquid nitrogen. RNAs were extracted using Trizol reagent according to the manufacturer's instructions (Invitrogen Corp., Carlsbad CA) and purified using the RNeasy kit, according to the manufactures instructions (Qiagen Corp., Valencia, CA). The quality and quantity of all purified RNA samples were assessed using agarose gel electrophoresis and the Nanodrop spectrophotometer (Nanodrop Technologies, Montchanin, DE). Eight μg of total RNA was labeled for each hybridization using the One-Cycle cDNA Synthesis Kit, according to the manufacturer's instructions (Affymetrix, Santa Clara CA) and sent to the University of Minnesota Microarray Facility for hybridization to the Affymetrix Maize GeneChip.

### Microarray data analysis

Affymetrix microarray data analysis was performed as described in [20]. Briefly, the GCOS software package v1.2 (Affymetrix) was used for signal acquisition and initial analysis. GeneSpring (Agilent Technologies, Palo Alto CA) software was used for GC-RMA (GC-content robust multi-array) processing of the .cel files that involved normalization between the arrays and a subsequent per gene normalization of the resulting values. Genes differentially expressed in DpDf plants relative to wild-type siblings were identified by performing a one-way ANOVA on the GC-RMA values using a parametric test with no assumption of equal variance. A Benjamin and Hochberg multiple testing correction was applied using a false-discovery rate significance threshold of 0.15, such that 15% of the genes identified in a test are likely to be falsely identified. A set of 727 genes were identified using this statistical test. The resulting list of the genes was further filtered to remove genes with very low expression levels or very small differences in expression between wild-type and DpDf genotypes since we had limited confidence that these genes were truly differentially expressed. This resulted in the removal of 60 genes with low expression values (GC-RMA expression values less than 50 units). Another 71 genes that with fold-change differences that were between 0.8 and 1.25 were also removed from the list of differentially expressed genes resulting in a filtered list of 596 genes. The GeneSpring software was used to perform hierarchical clustering analyses using a Pearson correlation method to create gene or condition trees based on specified gene lists, conditions and genotypes.

### Validation of gene map position and RT-PCR gene expression studies

Primers were designed using Primer 3.0 software [31] for a subset of the differentially expressed genes based on corresponding TC contigs [32] and MAGIs [33] and are listed in the Table SOM1. Bioinformatically predicted mapping positions were validated using oat × maize chromosome addition and radiation hybrid lines [18,21]. For gene expression validations, RNA samples used for cDNA synthesis were DNase treated (Promega, Madison, WI) and reverse transcribed using Superscript III reverse transcriptase (Invitrogen, Carlsbad, CA), according to the manufacturer's instructions. PCR reactions were performed on genomic DNA in a 15 μl total volume containing approximately 50 ng of DNA, 2 pmol of each primer, 0.4 units of HotStar Taq polymerase (Eppendorf, Westbury, NY), 1.58 μl of 10× reaction buffer, and 0.1 μl of 50 mM dNTPs. Cycling conditions of the PCR reactions were as follows: 94° for 15 min, 35 cycles of 94° for 30 sec, 60° for 30 sec, 72° for 90 sec, followed by 72° for 5 min. Amplified products were separated in a 1% agarose TBE gel and visualized by ethidium bromide staining. The concentrations of cDNAs were calibrated and made approximately equal by amplifying the maize *enhancer of zeste1 *(*Mez1 *– AF443596) gene that is expressed at approximately the same level in all genotypes analyzed.

### Knotted-like gene analysis

Gene-specific primers for nine knotted-like maize genes were derived from [34] and are listed in Table SOM1. A partial fragment of the *knox10 *sequence was kindly provided by Sarah Hake and Randall Kerstetter (personal communication). Additional sequence was identified by using GenomeWalker Universal kit (Clontech, Mountain View, CA) according to manufacture's recommendations with *knox10 *gene-specific primers starting with primers gR1 and gF1 [see Additional file 4 for primer sequences]. The genomic sequence was then used to design primers for amplification and sequencing of the cDNA from B73 seedling tissue.

### Bioinformatics analysis

Annotations for differentially expressed genes were based on information available at the TIGR Maize Gene Index [32]. The gene ontology (GO) annotations were obtained based on the assignment of the best *Arabidopsis *hits from the TAIR website [35]. The genetic map positions for Affymetrix array probe sets were predicted based on identity with genetically mapped sequences or inferred based upon identity with BAC contig sequences that contained genetically mapped markers [19,36,37].

### Phenotypic characterization of the DpDf plants

Families that segregated for first generation DpDf plant (derived from self-pollination of T5-6b heterozygotes) or later generation DpDf plants (derived from self-pollination of DpDf plants) were grown in the field season of summer 2006. Plant height, stalk diameter, severity of knotting on leaves, tassel length and thickness, and tassel viability were scored for 100 first generation DpDf plants, 100 later generation DpDf plants and 100 wild-type sibling plants at the stage when plants were flowering. Leaf knotting was scored on the scale of 0 to 5 with 5 being the most severe phenotype based on the number of knots on the leaf, number of leaves affected, and the distribution of knots. Tassel viability was scored on the scale of 0 to 5 with 5 being completely viable based on the number of affected branches and the length of the necrotic portion of the tassel. Stalk diameter was measured as the thickest diameter of the internode immediately above the uppermost ear node. Plant height was measured as the distance from the soil to the tip of the main tassel rachis.

## Authors' contributions

IM carried out the microarray studies, molecular biology and phenotypic assays. RLP and NMS conceived of the study, and participated in its design and coordination and helped to draft the manuscript. All authors read and approved the final manuscript.

## Supplementary Material

Additional file 1 **Annotation and identification of 596 genes that are differentially expressed in DpDf plants compared to their wild type siblings**. This file is available as an Excel spreadsheet that lists the differentially expressed genes and provides annotation, map position and accession numbers for each gene. Click here for file

Additional file 2 **Map positions for each of the Affymetrix probe sets**. This file is available as an Excel spreadsheet that lists the predicted map positions for ~7,000 features present on the maize Affymetrix microarray. Click here for file

Additional file 3 **Analysis of 334 genes that map to trisomic region of chromosome 5S**. This file is available as an Excel spreadsheet that lists 334 genes present in the trisomic region of chromosome 5. For each gene we have indicated the expression level in DpDf and wild-type plants as well as the annotation and map position. Click here for file

Additional file 4 **The list of primers used in genotyping, knox genes studies, validation of map position, and RT-PCR validation of gene expression**. An Excel spreadsheet file is provided listing each primer sequence used for this study. Click here for file
